# Fluorescence staining of the nucleus in living plant cells using dimidium bromide

**DOI:** 10.5511/plantbiotechnology.25.0508a

**Published:** 2025-12-25

**Authors:** Shintaro Ichikawa, Miho Kitamura, Yutaka Kodama

**Affiliations:** 1 Center for Bioscience Research and Education, Utsunomiya University, Tochigi 321-8505, Japan; 2 Graduate School of Regional Development and Creativity, Utsunomiya University, Tochigi 321-8505, Japan

**Keywords:** confocal microscopy, dimidium bromide, imaging, nucleolus, nucleus

## Abstract

Nuclear staining using fluorescent dyes is crucial for cytological studies in plants. However, few fluorescent dyes are suitable for live-cell imaging of the nucleus. Here, we demonstrate that dimidium bromide (DimBr), a commercially available fluorescent dye, can be used to stain the nucleus (nucleolus and nucleoplasm) in living plant cells. DimBr emits peak fluorescence at 600 nm at an excitation wavelength of 525 nm, making it well suited for use with green fluorescent protein. DimBr staining can be used in various plant species and allows time-lapse observation of the nucleus. Therefore, DimBr can be used to visualize the nucleus in living plant cells, making it a valuable tool for plant cell biology.

## Introduction

In eukaryotic organisms, the nucleus contains chromosomes, as well as membraneless structures such as the nucleolus and Cajal bodies (Kalinina et al. 2018; Taliansky et al. 2023). During various cellular processes, the nucleus in plant cells physically interacts with other organelles and changes its position in response to light and temperature (Higa et al. 2014; Jan et al. 2022; Liu and Li 2019; Ogasawara et al. 2013). Therefore, visualizing the subcellular localization of the nucleus is a key factor in studying organelle communication and relocation.

Due to their ease of use and availability, fluorescence staining methods are widely used to visualize the nucleus. Fluorescent probes including 4′,6-diamidino-2-phenylindole (DAPI), SYBR Green, and propidium iodide (PI) have been used in many studies (Barrell and Grossniklaus 2005; Hayashi et al. 2013; Kapuscinski 1995), but most of these dyes are used to stain chemically fixed cells, not live cells. When examining live cells, lengthy treatment with these dyes is required to stain the nucleus. On the other hand, the use of the *N*-aryl pyrido cyanine dye Kakshine and its derivatives allows simple and clearer observation of the nucleus in live cells (Uno et al. 2021). While Kakshine dyes can be employed for live-cell imaging, the development of additional dyes for staining the nucleus in living plant cells could contribute to studies of plant organelles.

Here, we report that dimidium bromide (DimBr), a commercially available fluorescent dye, can be used to visualize the nucleus in living plant cells. The DimBr staining procedure is simple and quick, taking roughly ten minutes, providing a new method for imaging and observing the nucleus in living plant cells.

## Materials and methods

### Plant materials and growth conditions

*Nicotiana benthamiana* seeds were sown on a soil mixture (vermiculite and potting mix; 2 : 1 [v/v]) and grown in white light (approximately 50 µmol photons m^−2^ s^−1^) under a 16-h-light/8-h-dark diurnal cycle at 25°C. Sterilized Arabidopsis (*Arabidopsis thaliana*, accession Columbia-0) seeds were sown on half-strength Murashige–Skoog medium containing 0.5% (w/v) gellan gum with 1% (w/v) sucrose, and 12-d-old seedlings were transferred to the soil mixture. Lettuce (*Lactuca sativa*) and cucumber (*Cucumis sativus*) seeds were sown on soil. Arabidopsis, lettuce, and cucumber were grown in soil under continuous white light (approximately 50 µmol photons m^−2^ s^−1^) at 22°C. The liverwort *Marchantia polymorpha* (accession Tak1) was grown in half-strength Gamborg’s B5 medium containing 1% (w/v) agar under the same continuous white light conditions at 22°C. Chinese hamster ovary (CHO)-K1 cells were cultured as previously described (Tanaka et al. 2019).

### DimBr staining

Leaves of 30- to 45-d-old *N. benthamiana* plants, 12- to 24-d-old Arabidopsis, 3-w-old lettuce, and 3-w-old cucumber were used. Leaf disks (2.0 mm in diameter) prepared using a hole puncher (KN-291-2, Natsume Seisakusho, Tokyo, Japan) were deaerated with 20 µM DimBr (Catalog No. D1815; Tokyo Chemical Industry) dissolved in water using a syringe and plunger and incubated in the same DimBr solution for 10–20 min before observation. CHO-K1 cells were treated with 20 µM DimBr in phosphate-buffered saline (PBS) for 10 min after removing the liquid culture medium. Before observation, the staining solution was removed, and the DimBr-treated cells were rinsed twice with PBS. A 10 mM stock solution of DimBr was prepared in water and stored at 4°C.

### Trypan blue staining

A solution of 20 µM DimBr was infiltrated into an *N. benthamiana* leaf using a syringe and plunger. As a control, water was infiltrated. After treating with DimBr for 1 h, trypan blue staining was performed according to a previous study (Fernández-Bautista et al. 2016). Briefly, the DimBr-treated leaf was immersed in a trypan blue staining solution [lactic acid : TE saturated phenol : glycerol : ultrapure water=1 : 1 : 1 : 1 (v/v) containing 10 mg ml^−1^ trypan blue] for 60 min. Images were taken after decoloring the leaf with ethanol.

### Plasmid construction

Primers used for plasmid construction are listed in Supplementary Table S1. To produce Arabidopsis Histone 2B (AT5G22880) fused with superfolder green fluorescent protein (H2B-sfGFP) (Pédelacq et al. 2006) as a nucleoplasm marker (Boisnard-Lorig et al. 2001), the DNA fragment encoding H2B was amplified by PCR using Arabidopsis cDNA as a template with the primers H2B-attB1-F and H2B-sfGFP-R (Supplementary Table S1). The DNA fragment encoding sfGFP was amplified by PCR using synthetic DNA (Fujii and Kodama 2015) as a template with the primers sfGFP-F and sfGFP-attB2-R (Supplementary Table S1). The two PCR products (*H2B* and *sfGFP* fragments) were fused by recombinant PCR with the primers H2B-attB1-F and sfGFP-attB2-R (Supplementary Table S1), generating the *H2B*-*sfGFP* fragment.

To produce Fibrillarin-sfGFP as a nucleolus marker (Koroleva et al. 2009), the DNA fragment encoding Fibrillarin (AT5G52470) was amplified by PCR using Arabidopsis cDNA as a template with the primers Fibrillarin-attB1-F and Fibrillarin-sfGFP-R (Supplementary Table S1). The *Fibrillarin* fragment was fused to the *sfGFP* fragment by recombinant PCR with the primers Fibrillarin-attB1-F and sfGFP-attB2-R (Supplementary Table S1), generating the *Fibrillarin*-*sfGFP* fragment.

To produce Coilin-sfGFP as a Cajal body marker (Collier et al. 2006), the DNA fragment encoding Coilin (AT1G13030) was amplified by PCR using Arabidopsis cDNA as a template with the primers Coilin-attB1-F and Coilin-sfGFP-R (Supplementary Table S1). The *Coilin* fragment was fused to the *sfGFP* fragment by recombinant PCR with the primers Coilin-attB1-F and sfGFP-attB2-R (Supplementary Table S1), generating the *Coilin-sfGFP* fragment.

The *H2B-sfGFP*, *Fibrillarin-sfGFP*, and *Coilin-sfGFP* DNA fragments were cloned into the pDONR207 or pDONR/Zeo vector by Gateway BP reaction (Invitrogen, Massachusetts, USA), generating pDONR207-H2B-sfGFP, pDONR207-Fibrillarin-sfGFP, and pDONR/Zeo-Coilin-sfGFP, respectively. The resulting plasmids and pDONR207-TIM21-Citrine, fluorescently labeling mitochondria with Citrine (Osaki and Kodama 2017), were recombined with the pGWB602 binary vector (Nakagawa et al. 2007), in which the cloned gene is driven by the cauliflower mosaic virus (CaMV) 35S promoter, by Gateway LR reaction (Invitrogen), generating pGWB602-H2B-sfGFP, pGWB602-Fibrillarin-sfGFP, pGWB602-Coilin-sfGFP, and pGWB602-TIM21-Citrine.

### Agroinfiltration

Agroinfiltration was performed as previously described with several modifications (Ichikawa et al. 2022). The pGWB602-H2B-sfGFP, pGWB602-Fibrillarin-sfGFP, pGWB602-Coilin-sfGFP, and pGWB602-TIM21-Citrine plasmids were introduced into Agrobacterium (*Rhizobium radiobacter* [syn. *Agrobacterium tumefaciens*]) strain GV2260, and the Agrobacterium transformants were cultured in 2 ml Luria–Bertani medium in a test tube at 28°C for 24 h in a shaking incubator. The cultured suspension was transferred to a tube and centrifuged at 4,000 g for 3 min. After removing the supernatant, the Agrobacterium pellet was resuspended in infiltration buffer (10 mM MgCl_2_, 10 mM MES-NaOH pH 5.7). The final suspension was adjusted to OD_600_=1.0. When simultaneously using two Agrobacterium strains, both suspensions were equally mixed. The resulting culture was infiltrated into the leaves of 30- to 45-d-old *N. benthamiana* plants. The leaf cells were observed 2 days after agroinfiltration.

### Confocal microscopy

Fluorescent DimBr, sfGFP, and chlorophyll signals were observed using an SP8X confocal microscope system (Leica Microsystems, Wetzlar, Germany). DimBr, sfGFP, and Citrine fluorescence were observed using hybrid detectors in photon counting mode and a water immersion lens (HC PL APO 63×/1.20 W CORR CS2, Leica Microsystems) for plant materials and oil immersion lens (HC PL APO 100×/1.40 OIL CS2, Leica Microsystems) for CHO-K1 cells. DimBr and chlorophyll fluorescence were excited with 488 nm light obtained from the white light laser (WLL) and detected at 600–650 and 680–720 nm, respectively. Sequential scan mode was used to detect fluorescence from both DimBr and sfGFP in the same cell, with sfGFP excited with 488 nm light and detected at 500–550 nm, and DimBr was then excited with 514 nm from the WLL and detected at 600–650 nm. DimBr, Citrine, and chlorophyll fluorescence were also detected using sequential mode; Citrine was excited with 514 nm light and detected at 525–560 nm, and DimBr and chlorophyll fluorescence were then excited with 488 nm from the WLL and detected at 600–650 and 680–720 nm, respectively. To prevent interference from chlorophyll fluorescence in the DimBr and sfGFP images, the time-gated method was used with a detection time of 0.5–1.2 ns (Kodama 2016).

Excitation and emission spectra of DimBr in the nucleus were measured every 5 nm using xyΛ and xyλ mode, respectively, installed in LAS X software (Leica Microsystems) by surrounding the fluorescence signal of DimBr in the nucleus with the region of interest tool.

A photostability test was performed using an excised *N. benthamiana* leaf rinsed for 10 min with water after DimBr staining. A WLL at 21% intensity (488 nm) was continuously irradiated to the leaf cells during a 60-min observation period. Images were captured at 10-min intervals.

For time-lapse imaging, the Arabidopsis specimen was mounted with TetraFix (Gellycle and Cosmo Bio) (Sakata et al. 2019). An argon laser of 0.2% intensity at 458 nm was used to irradiate part of the cell, and images were taken at 10-s intervals.

### Fluorescence intensity measurement

The fluorescence intensity of DimBr mixed with nucleic acids (DNA and RNA) was measured using an F2700 fluorescence spectrophotometer (Hitachi High-Tech). pDONR207-H2B-sfGFP plasmid DNA was used as the DNA, and DNase I (Takara Bio, Kusatsu, Japan)-treated total RNA from Arabidopsis was used as the RNA. A 5 µg DNA or RNA sample was added to 2 µM DimBr to prepare 500 µl of DNA or RNA solution, and the fluorescence intensity of 450 µl of each solution was measured; 20 mM HEPES-KOH pH 7.2 was used as the solvent. The fluorescence intensity of DimBr with DNA or RNA was detected at wavelengths between 510 and 750 nm with 488 nm excitation.

## Results

### DimBr staining of *N. benthamiana* can be used to visualize cell nuclei

**Figure 1:**
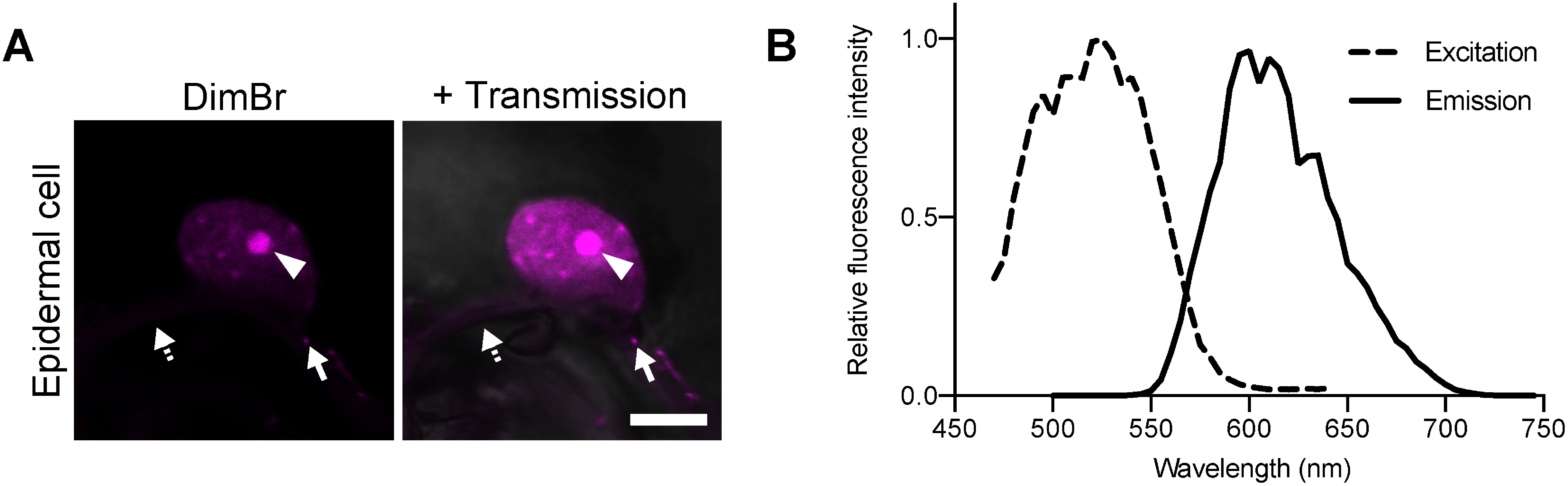
Figure 1. Fluorescence staining of the nucleus in *N. benthamiana* leaves using DimBr. (A) Epidermal cell treated with DimBr. A detached leaf disk (2.0 mm in diameter) created using a hole puncher was deaerated and stained with 20 µM DimBr for 10 min. Arrowheads indicate the brighter, smaller structure within the nucleus; solid and dashed arrows indicate punctate and cytosolic fluorescence signals, respectively. Scale bar, 5 µm. (B) Excitation and emission spectra of DimBr within the nucleus in epidermal cells. Measurement was performed on 10 nuclei, and the mean values were used to construct the graph. Each spectrum was normalized using the peak values of the excitation and emission spectra.

We previously demonstrated that commercially available fluorescent dyes can be used to visualize sub-organellar compartments in plants, such as the chloroplast outer envelope membrane and starch granules (Ichikawa et al. 2022, 2024). To identify fluorescent dyes suitable for labeling subcellular structures in living plant cells, we screened over 100 commercially available fluorescent dyes. When *N. benthamiana* leaves were treated with 20 µM DimBr for 10 min, the nucleus was clearly visible in epidermal cells, along with a brighter, smaller structure within the nucleus (Figure 1A). In addition, punctate fluorescence signals were detected in the cytoplasmic region (Figure 1A).

To identify the DimBr fluorescence spectrum in planta, we measured the fluorescence excitation and emission spectra in *N. benthamiana* leaf-epidermal cells stained with 20 µM DimBr. The spectra showed a wide Stokes shift, with peak excitation and emission wavelengths of 525 and 600 nm, respectively (Figure 1B). This suggested that DimBr could be used for in planta co-localization analysis with sfGFP, a bright GFP (Fujii and Kodama 2015) with a maximum excitation wavelength of 488 nm and an emission wavelength of 510 nm (Pédelacq et al. 2006).

As for the staining property, the photobleaching rate of DimBr in plant cells was verified. DimBr fluorescence was not photobleached during the continuous irradiation of the excitation laser for 60 min (Supplementary Figure S1A, B). These data indicate the strong photostability of DimBr in planta. Additionally, we tested the cytotoxicity of DimBr using trypan blue dye staining of dead or damaged cells. An *N. benthamiana* leaf was treated with DimBr for 1 h, followed by trypan blue staining. No strong blue signal was observed in the DimBr-treated region, similar to the water-treated region (Supplementary Figure S2), indicating that 1-h treatment of DimBr does not affect the cell viability.

### DimBr stains both the nucleoplasm and nucleolus

**Figure 2:**
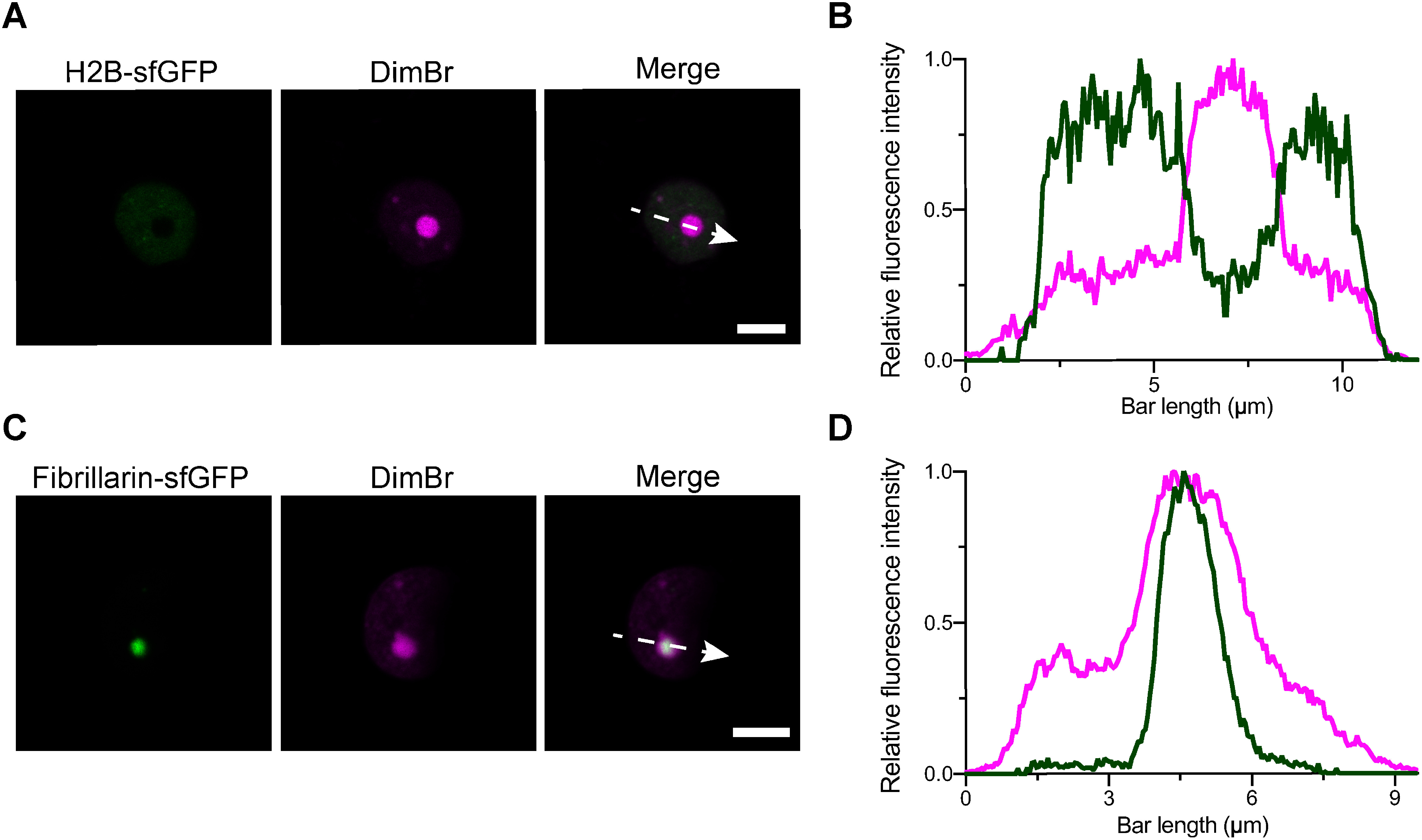
Figure 2. DimBr staining of the nucleoplasm and nucleolus. (A) Co-localization analysis of DimBr and H2B-sfGFP signals. The direction of the dashed arrow indicates the direction of measurement. Scale bar, 5 µm. (B) Fluorescence intensity of DimBr and H2B-sfGFP along the dashed arrow (A) measured with LAS X software in Quantify mode. The fluorescence intensities of DimBr and H2B-sfGFP were normalized using the peak value of each spectrum. (C) Co-localization analysis of DimBr and Fibrillarin-sfGFP. The direction of the dashed arrow indicates the direction of measurement. Scale bar, 5 µm. (D) Fluorescence intensity of Fibrillarin-sfGFP and DimBr along the dashed arrow (C) measured using LAS X software. The fluorescence intensities of DimBr and Fibrillarin-sfGFP were normalized using the peak value of each spectrum.

To identify the localization of DimBr fluorescence signals in the nucleus, we transiently expressed H2B-sfGFP as a nucleoplasm marker (Boisnard-Lorig et al. 2001) or Fibrillarin-sfGFP as a nucleolus marker (Koroleva et al. 2009) in *N. benthamiana* leaf-epidermal cells and stained the marker-expressing cells with 20 µM DimBr. H2B-sfGFP and Fibrillarin-sfGFP showed modest and strong fluorescence signals, respectively, both of which overlapped with DimBr fluorescence (Figure 2A, C). Profile analysis confirmed the co-localization of the nucleoplasm and nucleolus markers with DimBr (Figure 2B, D). These data indicate that DimBr stains both the nucleoplasm and nucleolus of nuclei in living *N. benthamiana* epidermal cells.

### Specificity of DimBr for staining nucleic acids

**Figure 3:**
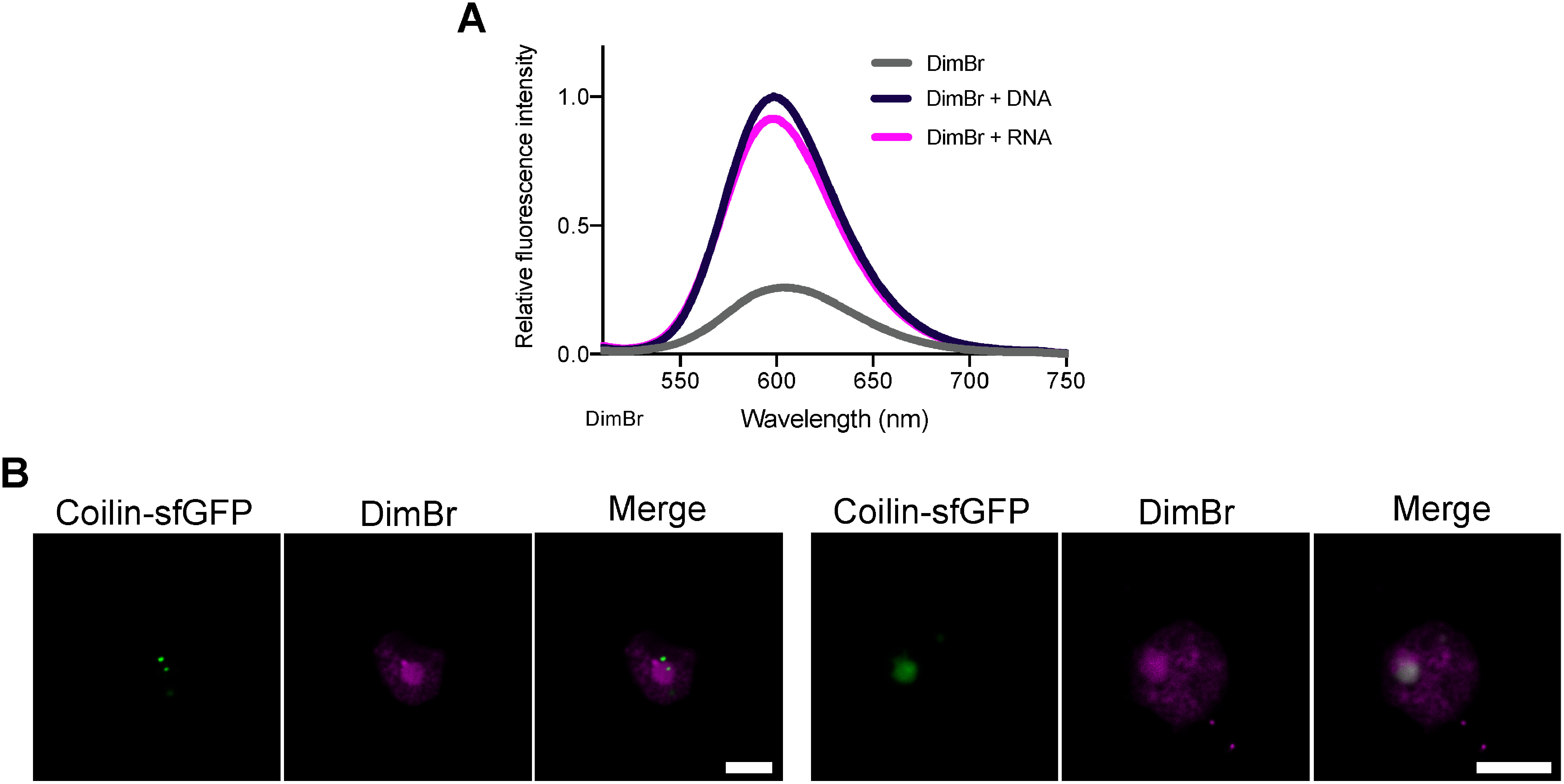
Figure 3. Binding of DimBr to DNA or RNA. (A) Relative fluorescence intensity of DimBr by itself or mixed with DNA or RNA. Plasmid DNA (pDONR207-H2B-sfGFP) and total RNA from Arabidopsis were used as DNA and RNA materials, respectively. The measurements were repeated three times, and the mean value was used to construct the graph. Each fluorescence intensity value was normalized using the peak value of the fluorescence intensity from the DimBr/DNA mixture. (B) Two representative images of Coilin-sfGFP in *N. benthamiana* leaf-epidermal cells stained with DimBr. Scale bars, 5 µm.

As observed in previous studies (Dougherty 1982; Hawkins and Willis 1969; Lerman 1964), DimBr fluoresced strongly in vitro when admixed with DNA or RNA (Figure 3A). To test whether DimBr stains other membraneless structures containing RNA within the nucleus, we transiently expressed Coilin-sfGFP, a Cajal body marker (Collier et al. 2006), in *N. benthamiana* leaf-epidermal cells and observed the cells after staining with 20 µM DimBr. Although the localization patterns of Coilin-sfGFP varied between cells, the fluorescence signal of DimBr did not completely co-localize with that of Coilin-sfGFP in most cells examined (Figure 3B). These results indicate that DimBr does not stain all RNA-containing structures within the nucleus.

### DimBr staining in different cell types and various plant species

**Figure 4:**
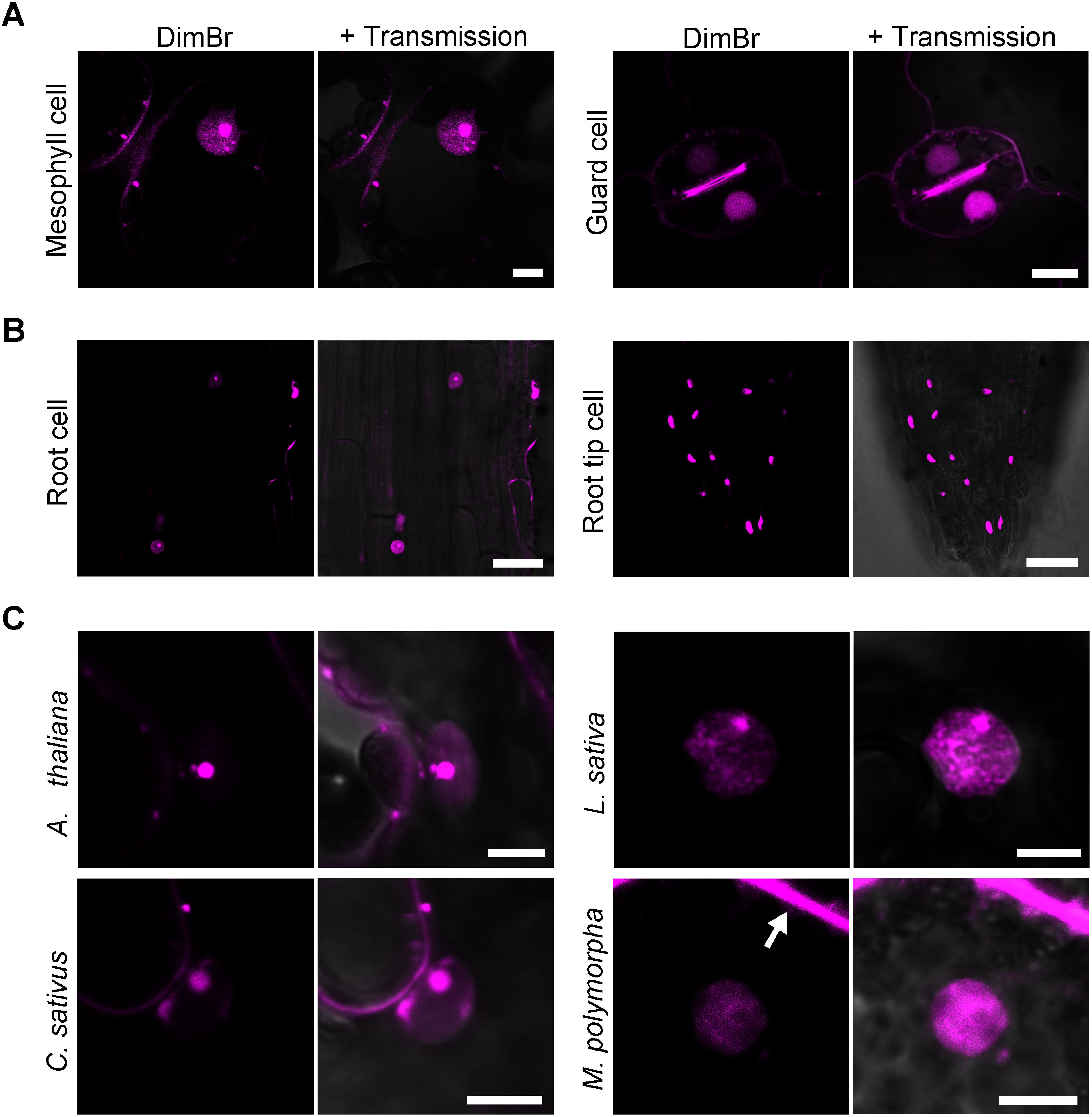
Figure 4. DimBr staining of multiple cell types from various plant species. (A) Mesophyll and guard cells of *N. benthamiana* leaves stained with 20 µM DimBr. Scale bars, 10 µm. (B) Root and root tip cells of *N. benthamiana* treated with 20 µM DimBr. Scale bars, 50 µm. (C) Epidermal cells of Arabidopsis (*A. thaliana*), lettuce (*L. sativa*), and cucumber (*C. sativus*) and gemmaling cells of liverwort (*M. polymorpha*) stained with 20 µM DimBr. In the liverwort image, the arrow points to the strong cytosolic fluorescence signal. Scale bars, 5 µm.

In addition to staining leaf-epidermal cells, DimBr was also able to stain mesophyll and guard cells in *N. benthamiana* leaves (Figure 4A). We detected DimBr staining of the nucleolus in mesophyll cells but not guard cells (Figure 4A). In addition, DimBr stained the nucleoplasm and nucleolus in root cells, but only the nucleoplasm in root tip cells (Figure 4B). Thus, the ability of DimBr to stain the nucleoplasm and/or nucleolus varies depending on the cell type.

To assess DimBr staining in other plant species, we observed Arabidopsis, lettuce, cucumber, and *M. polymorpha* cells stained with 20 µM DimBr (Figure 4C). In leaf-epidermal cells of Arabidopsis, lettuce, and cucumber, DimBr stained both the nucleoplasm and nucleolus (Figure 4C). In *M. polymorpha* mesophyll cells, DimBr staining allowed us to observe the nucleus and produced strong cytosolic fluorescence signals (Figure 4C). These data suggest that DimBr can be used to visualize the nucleus in multiple plant species and that the staining pattern varies depending on the species.

Moreover, we verified if DimBr staining can be used in cells of other organisms such as animals. When Chinese hamster ovary (CHO)-K1 cells were subjected to DimBr staining, the nucleolus and cytosol, but not the nucleoplasm, were stained (Supplementary Figure S3). These data suggest that DimBr can stain the nucleolus of both plants and animals, but staining of nucleoplasm and cytosol varies between organisms.

### DimBr staining enables live-cell imaging of nucleus relocation

**Figure 5:**
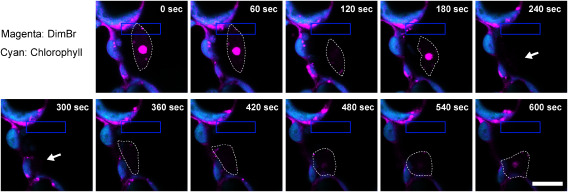
Figure 5. Snapshots of time-lapse imaging of nucleus relocation in Arabidopsis. Images were extracted from Supplementary Video S1. A blue laser (458 nm) was used to irradiate the area within the blue-lined rectangle. Dashed lines indicate the outline of the nucleus, and arrows indicate the putative position of the framed nucleus. The decrease in DimBr fluorescence intensity at the nucleus is due to an out-of-focus observation because DimBr has a strong photostability (Supplementary Figure S1). Scale bar, 10 µm.

To test whether DimBr can be used effectively for live-cell imaging, we performed time-lapse observation of nucleus movement in response to strong blue light (Higa et al. 2014). When an Arabidopsis leaf-epidermal cell stained with 20 µM DimBr was partially irradiated by blue laser at 458 nm for 10 min, the DimBr-visualized nucleus was seen to avoid the laser-irradiated area (Figure 5, Supplementary Video S1), indicating that the behavior of the nucleus can be tracked in living plant cells stained with DimBr.

## Discussion

We have developed a rapid, easy DimBr-based method for observing nuclei in living plant cells. Among fluorescent dyes used to stain the nucleus, DAPI, SYBR Green, and PI do not allow live-cell imaging, whereas DimBr and Kakshine dyes can be used to perform time-lapse observation of the nucleus (Figure 5, Supplementary Video S1) (Uno et al. 2021). Kakshine dyes have higher binding specificity for DNA (Uno et al. 2021), while DimBr equally binds to both DNA and RNA (Figure 3A). This binding property of DimBr would allow us to visualize both nucleoplasm and nucleolus. A previous study demonstrated that DimBr-treated amebae divide exponentially, but their growth rate declines 3–4 days after DimBr treatment due to the inhibition of DNA and RNA synthesis (Hawkins and Willis 1969), suggesting that DimBr staining would not be suitable for long-term observation. Given that we successfully performed time-lapse imaging of the DimBr-stained nucleus during a 10-min period (Figure 5, Supplementary Video S1), however, DimBr-based nucleus imaging should be available for at least several minutes after staining.

DimBr emits red fluorescence with a peak at 600 nm when excited at a maximum wavelength of 525 nm in living cells (Figure 1B). This DimBr spectrum property makes it easy to simultaneously use DimBr along with other appropriate fluorescent proteins, particularly short-wavelength fluorescent proteins such as GFP (Griesbeck et al. 2001; Heim et al. 1994, 1995). Note that Kakshine dye variants (PC1, PC2, or PC3), which can be also used in living cells, may have similar spectral properties (excitation peak at approximately 530–560 nm and emission peak at approximately 550–600 nm) (Uno et al. 2021). In the present study, we used sfGFP with DimBr (Figure 2, 3B). The excitation and emission spectra of DimBr (Figure 1B) indicate that orange fluorescent proteins, including monomeric Kusabira-Orange 2 (mKO2), could be used with DimBr (Fujii et al. 2018; Sakaue-Sawano et al. 2008). However, the use of red fluorescent proteins such as monomeric red fluorescent protein (mRFP1) with DimBr should be avoided due to their close emission spectra (Campbell et al. 2002).

DimBr has a similar structure to ethidium bromide (EtBr), which contains an acridine skeleton. Acridine dyes intercalate DNA and RNA (Lerman 1964), suggesting that, like EtBr, DimBr is likely to have a carcinogenic effect. According to safety data sheets from several manufacturers (e.g., Tokyo Chemical Industry), the acute toxicity (dermal and inhalation) of DimBr is classified as category 4 in the globally harmonized system (GHS) criteria (Winder et al. 2005). No classification in regard to acute toxicity (dermal) is listed for EtBr, but its acute toxicity (inhalation) is classified as category 1, the most dangerous category (Winder et al. 2005). This information supports the “Warning” and “Danger” labels used for DimBr and EtBr, respectively, in the GHS criteria (Winder et al. 2005). Furthermore, because EtBr is more membrane permeable than DimBr (Lalchhandama 2016; Watkins 1952), EtBr is expected to be more harmful. Nevertheless, it is important to note that care should be taken when using DimBr (e.g., wearing rubber gloves).

In the present study, after DimBr staining, we observed cytosolic fluorescence signals in plant cells (Figure 1). DimBr fluorescence was stronger when admixed with DNA or RNA but still visible in their absence (Figure 3A), indicating that DimBr accumulates in the cytosol even when not bound to nucleic acids. We also detected punctate DimBr signals in the cytoplasmic region (Figure 1). Since the punctate DimBr signals were observed within mitochondria and chloroplasts (Supplementary Figure S4), genomic DNA and/or structures containing RNA within these organelles might be stained by DimBr. In addition to organellar nucleic acids, several protein-RNA complexes (e.g., stress granules) are reported to exist in the cytoplasmic region (Chantarachot and Bailey-Serres 2018; Weber et al. 2008), suggesting that DimBr might stain RNA-containing complexes in the cytoplasm, making them appear as punctate signals. As DimBr is a cationic compound, it might also bind anionic compounds other than DNA and RNA. A previous study reported that cationic EtBr can bind to anionic sodium dodecyl sulfate, but not to cationic and neutral surfactants (Pal et al. 1998). Taken together, several types of structures and/or compounds might be detected as background fluorescence signals in DimBr staining.

Although DimBr-bound DNA and RNA showed comparable fluorescence intensities (Figure 3A), the fluorescence intensity from the nucleolus was stronger than that from the nucleoplasm (Figure 2A, B). Due to the tight association of genomic DNA and histones in chromatin, it might be difficult for DimBr to access genomic DNA in living animal cells, as observed in a previous study using EtBr (Stockert 1974). This previous study supports our observation that DimBr stains only the nucleolus and cytosol in CHO-K1 cells (Supplementary Figure S3), which may be explained by the difference in DNA-histone association between plant and animal cells. Unlike genomic DNA associated with histones, DimBr might readily access RNA (e.g., free RNA) in the nucleolus, allowing strong DimBr fluorescence signals to be observed in the nucleolus (Figure 3B). If this is indeed the case, the different staining results between the nucleolus and Cajal bodies suggest that more free RNA is contained in the nucleolus than in Cajal bodies (Figure 2C, 3B). When we observed guard cells and root tip cells stained with DimBr, only the nucleoplasm, not the nucleolus, was observed (Figure 4A, B). The differential accessibility of DimBr to DNA or RNA within the nucleus might also explain the staining patterns in guard cells and root tip cells.

In this study, we discovered that DimBr can be used to visualize the nucleus (nucleoplasm and nucleolus) in living plant cells. We recently explored the use of commercially available fluorescent dyes for staining subcellular structures in many types of plants (Ichikawa et al. 2022, 2024). Further exploration of the use of fluorescent dyes to stain subcellular structures would help advance the study of plant cell biology and biotechnology in various living plant species.

## Abbreviations

CaMV: cauliflower mosaic virus
CHO: Chinese hamster ovary
DAPI: 4′,6-diamidino-2-phenylindole
DimBr: dimidium bromide
EtBr: ethidium bromide
GHS: globally harmonized system
mKO2: monomeric Kusabira-Orange 2
mRFP1: monomeric red fluorescent protein
PBS: phosphate-buffered saline
PI: propidium iodide
sfGFP: superfolder green fluorescent protein
WLL: white light laser

## Accession numbers

Sequence information was obtained from TAIR (https://www.arabidopsis.org (Accessed Jun 1, 2023)) using the following accession numbers: H2B (AT5G22880), Fibrillarin (AT5G52470), Coilin (AT1G13030), and TIM21 (AT4G00026).

## Description of supplementary files

Supplementary Table S1. Primers used in this study.

Supplementary Figure S1. Photobleaching analysis of DimBr staining in leaves.

Supplementary Figure S2. Viability test of DimBr staining in leaves.

Supplementary Figure S3. DimBr staining in CHO-K1 cells.

Supplementary Figure S4. Organellar nucleic acid staining using DimBr.

Supplementary Video S1. Time-lapse observation of nucleus relocation in response to a blue laser.
