# Supplementary material for: Fluorescence staining of the nucleus in living plant cells using dimidium bromide
Source: Plant Biotechnol (Tokyo). 2025 Dec 25;42(4):441–8. doi: 10.5511/plantbiotechnology.25.0508a (PMC12781915; doi:10.5511/plantbiotechnology.25.0508a)
Supplement: Supplementary Data [file plantbiotechnology-42-4-25.0508a-s001.pdf]

Supplementary Table S1. Primers used in this study.

Primer name	Primer sequence (5’ to 3’ direction)
H2B-attB1-F	GGGGACAAGTTTGTACAAAAAAGCAGGCTTCATGGCGAAGGCAGATAAG
H2B-sfGFP-R	GCCCTTGCTCACCATAGAACTCGTAAACTTCGT
sfGFP-F	ATGGTGAGCAAGGGCGAGGAGCTGTTCACC
sfGFP-attB2-R	GGGGACCACTTTGTACAAGAAAGCTGGGTCTCACTTGTACAGCTCGTCC
Fibrillarin-attB1-F	GGGGACAAGTTTGTACAAAAAAGCAGGCTTCATGAGACCCCCAGTTACA
Fibrillarin-sfGFP-R	CTCGCCCTTGCTCACCATTGAGGCTGGGGTCTTTTG
Coilin-attB1-F	GGGGACAAGTTTGTACAAAAAAGCAGGCTTCATGGAGGAAGAGAAGGTG
Coilin-sfGFP-R	CTCGCCCTTGCTCACCATAATCTCTTTCTGAGATCT

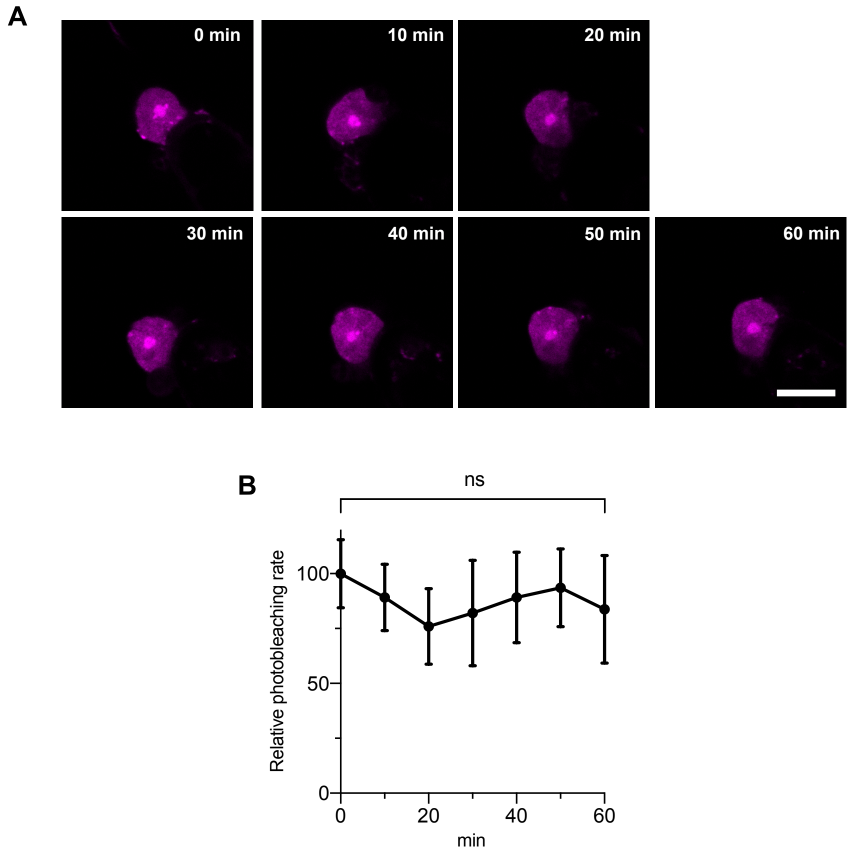

Supplementary Figure S1. Photobleaching analysis of DimBr staining in leaves. (A) Photostability test of DimBr in the *N. benthamiana* nucleus. Excitation laser at 488 nm was continuously irradiated to the DimBr-stained nucleus during 60-min observation period. Scale bar, 10 µm. (B) Measurement of the photobleaching rate of DimBr using the images in (A). After quantifying the fluorescence intensity of the nucleus, normalized average and standard deviation were plotted. The quantification was performed with 6 nuclei images. There is no significant difference between the photobleaching rate of 0-min and 60-min laser irradiation (student's t-test, $p>0.05$).

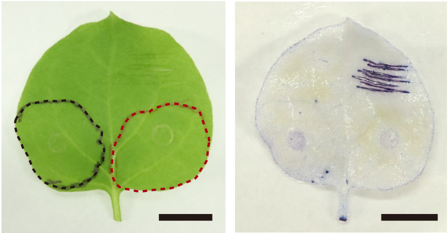

Supplementary Figure S2. Viability test of DimBr staining in leaves. Trypan blue staining using an *N. benthamiana* leaf treated with water or DimBr. As a positive control, a part of the leaf was wounded using a razor blade. Black and red circles indicate the water- and DimBr-infiltrated regions, respectively. Scale bar, 1 cm.

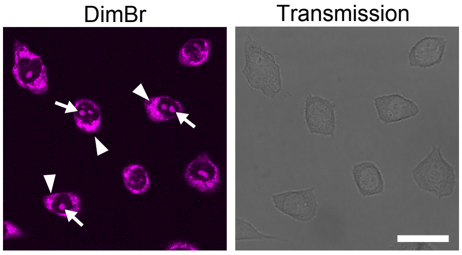

Supplementary Figure S3. DimBr staining in CHO-K1 cells. CHO-K1 cells were treated with 20 μM DimBr in PBS for 10 min. Arrows and arrowheads indicate DimBr signal at of the nucleolus and cytosol, respectively. Scale bar, 25 μm.

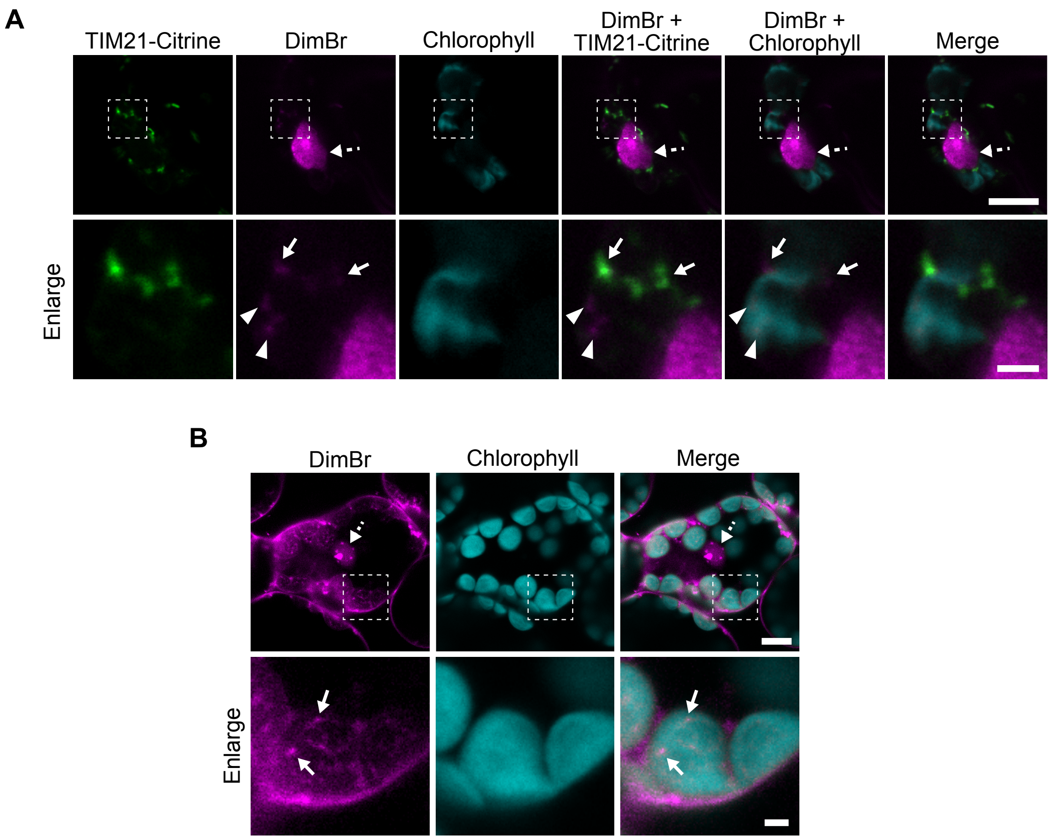

Supplementary Figure S4. Organellar nucleic acid staining using DimBr. (A) Observation of DimBr, TIM21-Citrine, and chlorophyll fluorescence in an *N. benthamiana* cell. Dashed arrows and squares indicate the positioning of the nucleus and the cropped region for the enlarged images, respectively. Very weak DimBr fluorescence was co-localized with TIM21-Citrine (arrows, mitochondria) and chlorophyll fluorescence (arrowheads, chloroplasts). Scale bar, 10 µm (original images) and 5 µm (enlarged images). (B) DimBr fluorescence within chloroplasts in Arabidopsis. Dashed arrows and squares indicate the positioning of the nucleus and the cropped region for the enlarged images, respectively. Arrows point to the punctate fluorescence signals of DimBr detected within chlorophyll fluorescence. Scale bar, 10 µm (original images) and 5 µm (enlarged images).
