# Supplementary material for: Fluorescence staining of the nucleus in living plant cells using dimidium bromide
Source: Plant Biotechnol (Tokyo). 2025 Dec 25;42(4):441–8. doi: 10.5511/plantbiotechnology.25.0508a (PMC12781915; doi:10.5511/plantbiotechnology.25.0508a)
Supplement: Supplementary Data [file plantbiotechnology-42-4-25.0508a-s003.docx]

**Supplementary Video S1.** Time-lapse observation of nucleus relocation in response to a blue light laser.
